# High Fat Diet Dysbiotic Mechanism of Decreased Gingival Blood Flow

**DOI:** 10.3389/fphys.2021.625780

**Published:** 2021-03-03

**Authors:** Dragana Stanisic, Nevena Jeremic, Suravi Majumder, Sathnur Pushpakumar, Akash George, Mahavir Singh, Suresh C. Tyagi

**Affiliations:** ^1^Department of Dentistry, Faculty of Medical Sciences, University of Kragujevac, Kragujevac, Serbia; ^2^Department of Physiology, School of Medicine, University of Louisville, Louisville, KY, United States; ^3^Department of Pharmacy, Faculty of Medical Sciences, University of Kragujevac, Kragujevac, Serbia

**Keywords:** gut microbiota, lipopolysaccharide, matrix metalloproteinases, Laser Doppler flowmetry, periodontal disease, *Lactobacillus rhamnosus*, blood flow

## Abstract

The gut microbiome has a very important role in human health and its influence on the development of numerous diseases is well known. In this study, we investigated the effect of high fat diet (HFD) on the onset of dysbiosis, gingival blood flow decreases, and the periodontal matrix remodeling. We established a dysbiosis model (HFD group) and probiotic model by *Lactobacillus rhamnosus* GG (LGG) treatment for 12weeks. Fecal samples were collected 24h before mice sacrificing, while short chain fatty acids (SCFA) analysis, DNA extraction, and sequencing for metagenomic analysis were performed afterwards. After sacrificing the animals, we collected periodontal tissues and conducted comprehensive morphological and genetic analyses. While HFD reduced *Bacteroidetes*, SCFA, and gingival blood flow, this type of diet increased *Firmicutes*, lipopolysaccharide (LPS) binding protein, TLR4, pro-inflammatory cytokines (TNF-α, IL-1β, and IL-6), matrix metalloproteinases (MMP-2 and MMP-9) expression, and also altered markers of bone resorption (OPG and RANKL). However, LGG treatment mitigated these effects. Thus, it was observed that HFD increased molecular remodeling *via* inflammation, matrix degradation, and functional remodeling and consequently cause reduced gingival blood flow. All of these changes may lead to the alveolar bone loss and the development of periodontal disease.

## Introduction

Changes in lifestyle influence usual diet habits and it is already established that consumption of diets with a high content of fats, proteins, and sugars modulate the gut microbiota and consequently affect the whole organism (Tremaroli and Bäckhed, 2012). Gastrointestinal tract presents one of the primary sites for bacterial colonization. More than 1,000 species of bacteria form intestinal flora which is primarily composed of anaerobes that make up more than 99% of gut microbes including *Firmicutes*, *Bacteroidetes*, *Proteobacteria*, *Archaebacteria*, and *Actinomycetes*. More than 90% of those mentioned are *Firmicutes* and *Bacteroidetes* (Zhang and Yang, 2016). The ratio of *Firmicutes* and *Bacteroidetes* differs depending on age evolves according to the different age group. For newborns, ratio of 0.4 was measured, 10.9 for adults, and 0.6 for the elderly (Mariat et al., 2009). High fat diet (HFD) leads to a reduction in *Bacteroidetes* and an increase in *Firmicutes* levels (Murphy et al., 2015). These changes have been implicated in a numerous diseases (Murphy et al., 2015), including periodontitis (Chen et al., 2017; Jia et al., 2019; Yu et al., 2019; Curtis et al., 2020). In addition, HFD increases the number of pathogenic bacteria in the gut, resulting in enhanced lipopolysaccharide (LPS) and lipopolysaccharide binding protein (LBP) production (Hersoug et al., 2018). Furthermore, HFD increases gut permeability, inflammatory response, and oxidative stress. At the same time, this diet decreases the production of short chain fatty acids (SCFA; Lu et al., 2018).

Moreover, HFD upsets the balance in good and bad gut bacteria, LPS production (Potter et al., 2016; Hersoug et al., 2018), decreases SCFA production (Lu et al., 2018), and affects blood vessels and blood flow (Wilde et al., 2000; Bui et al., 2010). Periodontium, gingiva, periodontal ligament, as well as alveolar bone, are greatly vascularized tissues. Adequate blood supply, as well as a healthy vascular endothelial system, is necessary for their function and nutrition (Nguyen et al., 2015; Veronesi et al., 2018; Komaki, 2019). Maintaining the equilibrium between matrix metalloproteinases (MMP) and their inhibitors in the tissues has a crucial role in keeping the endothelial system in good condition (Donnini et al., 2004; Rodrigues and Granger, 2015). Disturbance in local vascular flow is in correlation with inhibition of tissue oxygenation and reduction of microvascular blood flow. Disorder of blood flow activity is noted to be an early marker for progression of microvascular pathology (Clough et al., 2017; Mitra et al., 2018; Ohsugi et al., 2019). HFD contributes to endothelial dysfunction and loss of integrity (Clough et al., 2017; Mitra et al., 2018; Münch et al., 2019) it has been shown that 2h after a fatty meal, HFD causes endothelial damage by vascular occlusion and leads to decreased blood flow (Bui et al., 2010; Münch et al., 2019). Reduced blood flow in periodontal tissue, especially in gingiva, can lead to periodontal disease.

According to available literature data, periodontal disease is a dysbiosis condition of the oral microbiome (Payne et al., 2019). Qualitatively and quantitatively altered oral microorganisms induce an enhanced immune response (Gao et al., 2018; Graves et al., 2019). Thus, modified microorganisms can cause systemic inflammation by reaching microorganisms in the gut thorough swallowing and hematogenously dissemination of proinflammatory cytokines [Interleukin-1 Beta (IL-1β), Interleukin-6 (IL-6), Tumor Necrosis Factor Alpha (TNF-α)] or bacteria. Gut dysbiosis causes an increase in the gut mucosa permeability resulting in endotoxemia and systemic inflammation (Torres et al., 2017) which consequently causes intensified bone resorption. The production of proinflammatory cytokines, TNF-α, IL-1β, Interleukin-17 (IL-17) promotes survival, proliferation, osteoclasts activity, and suppression of osteoblasts activity. The cytokines IL-1β, TNF-α, and IL-17 cause osteoblasts to enhance the receptor activator of nuclear factor kappa-*Β* ligand (RANKL) release. RANKL binds to RANK on osteoclasts precursors and promotes osteoclastogenesis. Increscent in number of osteoclasts leads to resorption of the bone (Hajishengallis, 2015; Torres et al., 2017). HFD also promotes the formation of periodontal disease (Li et al., 2015; Virto et al., 2018a) *via* an increase in cytokine levels and alveolar bone loss (Fujita and Maki, 2016; Virto et al., 2018a,b). Virto et al. (2018a) indicated an increase in alveolar bone loss by 27.71% (2.28μm) in the HFD group compared to the control group.

**Figure 1:**
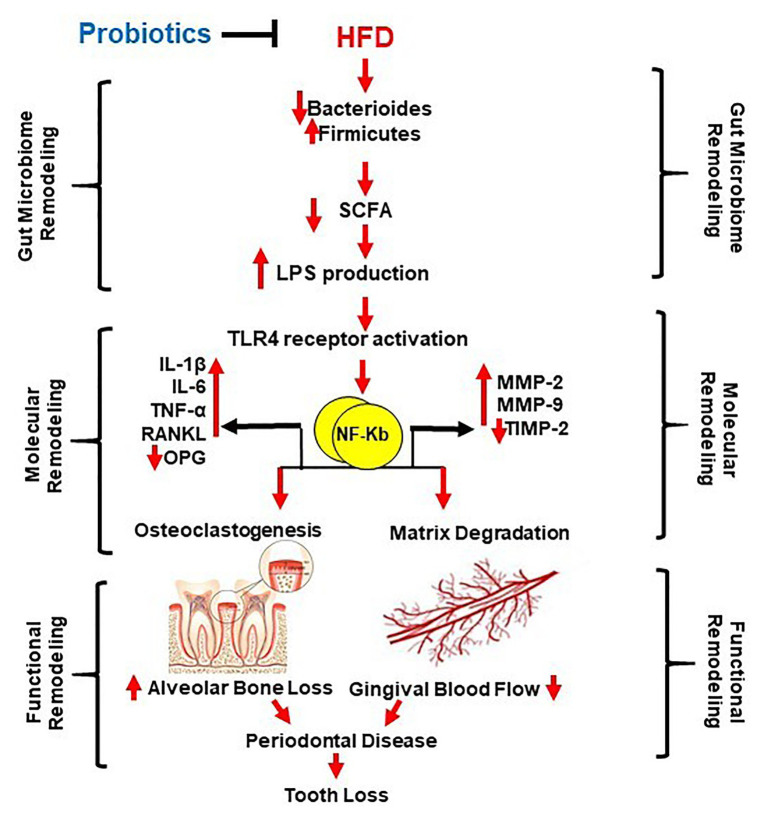
Schematic representation of the study aim. High fat diet (HFD) causes gut dysbiosis (*via* decreased short chain fatty acids (SCFA) and Bacteroides, and increased Firmicutes and Proteobacteria) resulting in increased lipopolysaccharide (LPS) production and LPS/TLR4 pathway activation in the oral cavity and gastrointestinal tract. Consequently, molecular remodeling occurs by increasing proinflammatory cytokines (IL-1β, IL-6, and TNF-α) and matrix metalloproteinases (MMP-2 and MMP-9). Due to the mentioned consequences, functional remodeling occurs, by an increase of proinflammatory cytokines, RANKL, and the decrease of OPG. Osteoclastogenesis and alveolar bone loss are promoted by disturbance in expression and activity of MMPs, matrix degradation, and gingival blood flow. This together cause periodontal disease and possibility of tooth loss. Probiotics maids mitigate all these consequences.

It would be very useful to improve and supplement the diet with probiotics with the aim of obtaining a healthy gut microbiota, prevention, and treatment of gastrointestinal diseases. This will also be beneficial in sense of maintaining gingival blood flow at basal level with consequent prevention of periodontitis development. *Lactobacillus rhamnosus* GG (LGG) is a Gram-positive rod-shaped facultative anaerobe, capable of surviving low levels of pH in the stomach and bile acids in the duodenum. It is one of the most used probiotics microorganisms in the world. Studies also proved its beneficial effect in the prevention and treatment of atopic dermatitis (Huang et al., 2017), hypercholesterolemia (Shimizu et al., 2015), and obesity (Sanchez et al., 2014). This probiotic does not ferment sucrose and lactose, so it has been suggested that this anaerobe could significantly reduce the risk of caries (Gruner et al., 2016; Jiang et al., 2018). Moreover, LGG possesses significant *in vivo* anti-inflammatory properties (Lin et al., 2009). However, its effect in the treatment of gingival blood flow and periodontitis has not been fully investigated. Given its non-cariogenic and anti-inflammatory properties, LGG turned out to be a good candidate in the prevention and treatment of periodontal disease (Gatej et al., 2018). The aim of this study was to prove the influence of HFD and LGG on the state of the gut microbiota, gingival blood flow, and the development of periodontal disease (Figure 1).

## Materials and Methods

### Animal Maintenance and Diet Protocol

Male wild-type (C57BL/6J) mice were purchased from the Jackson Laboratory (Bar Harbor, ME). All animals were 8weeks-old and maintained in 12:12h light-dark cycle in the animal facility of the University of Louisville. All animal protocols and care were carried out according to the guidelines of the National Institute of Health (NIH) and were approved by the Institutional Animal Care and Use Committee (IACUC #19592) of the University of Louisville, KY.

Animals were divided into four experimental groups: (1) Wild-type C57BJ/L6 mice (WT), (2) HFD-supplemented wild-type mice (HFD), (3) LGG-supplemented wild-type mice (LGG), and (4) HFD and LGG-supplemented wild-type mice (HFD + LGG; *n* = 5/groups). To create dysbiosis condition, the mice were fed with an HFD (TD.88137; Harlan Laboratories Inc., Indianapolis, IN, United States) for a period of 12weeks, and with or without oral probiotic – LGG @ 2.5 × 10^5^CFU for a period of 12weeks in drinking water (Gatej et al., 2018; He et al., 2018; George et al., 2020). Mice in the control group were fed with the standard chow diet. The standard diet contained 20% protein, 53% starch, 9% fat, and 5% fiber, while the HFD contained 15.3% protein, 42.7% starch, and 42.0% fat (TD.88137; Harlan Laboratories Inc., Indianapolis, IN, United States). Appropriate diets (standard or HFD, depending on the group) and water are provided *ad libitum*.

### Measurement of Blood Glucose Levels

Glucose levels in all examined groups were measured according to manufacturer’s instructions for commercially available kit (OneTouch Ultra2, LifeScan, Inc).

### Gingival Blood Flow Measurement

Gingival blood flow was measured as it was previously described, in break before procedure animals were under an intraperitoneal combination of anesthetics: ketamine (Ketamine 80mg/kg) and xylazine (Xylazine; 10mg/kg). The gingival blood flow was measured by Laser Doppler using Speckle Contrast Imager (Moor FLPI, Wilmington, DE) at room temperature. The camera (580 9 752 resolution) was positioned 15cm from the buccal surface of the gingiva (Clarke and Shephard, 1984; Baab and Oberg, 1987; George et al., 2018).

### Microbiota Analysis

Fecal samples were collected 24h before animal sacrifice and subsequently analyzed at Microbiome Insights Inc.; Vancouver, BC V6R 4K6; Canada. The following analyzes were performed to detect gut dysbiosis: analysis of SCFA, Deoxyribonucleic acid (DNA) extraction and sequencing, and sample preparation according to an already existing database [DNA Extraction + Library preparation and sequencing on the Illumina Next Seq (2× 150 BP); Zhao et al., 2006; Kozich et al., 2013; Bolger et al., 2014; Clarke et al., 2019; Wood et al., 2019].

### Measurement of LBP

Determination of LBP concentration from serum was archived by using ELISA kit with following all already established manufacturer’s protocol. We used Mouse LBP ELISA Kit (ab213876, Sigma-Aldrich, St. Louis, Missouri, United States; Chivero et al., 2019).

### Gelatin Zymography Analysis

Activity of MMP was measured using gelatin gel zymography as described previously (Stanisic et al., 2020). For this analysis, we used samples of periodontal tissue and the gels were stained with the Coomassie R-250 brilliant blue dye. The clear digested regions represent MMP activity which were quantified densitometrically using the Un-Scan-It software (Silk Scientific Inc., Orem, UT).

### RT-PCR Analysis

Real-time polymerase chain reaction (RT-PCR) analysis was conducted as it is previously described (Livak and Schmittgen, 2001; George et al., 2018).

We used the right mandibula (periodontal tissue; *n* = 5 per group) which were homogenized in a solution of TRIzol reagent (cat no. 15596018; Invitrogen; Thermo Fisher Scientific, Inc., Waltham, MA, United States) The sequences of the primers are shown in Table 1.

**Table 1:** Primers used for RT-PCR.

Target gene	Forward primer sequences (5'-3')	Reverse primer sequences (5'-3')
TLR4	CAA GGG ATA AGA ACG CTG AGA	GCA ATG TCT CTG GCA GGT GTA
IL-1β	GAAATGCCATTTGACAGTG	CTGGATGCTCTCACTAGGACA
IL-6	CTGCAAGAGACTTCCATCCA	CAGGTCTGTTGGGAGTGGT
TNF-α	CATCTTCTCAAATTCGAGTGACAA	TGGGAGTAGACAAGGTACAACCC
MMP-2	CTGATGGCACCCATTTACACCT	GATCTGAGCGATGCCATCAAA
MMP-9	AGAGATGCGTGGAGAGTCGAA	AAGGTTTGGAATCTGCCCAGG
TIMP-2	CAAGTTCTTCGCCTGCATCAA	TCGAAACCCTTGGAGGCTT
RANKL	CAGCATCGCTCTGTTCCTGTA	CTGCGTTTTCATGGAGTCTCA
OPG	ACCCAGAAACTGGTCATCAGC	CTGCAATACACACACTCATCACT
GAPDH	AGGTCGGTGTGAACGGTTTG	TGTAGACCATGTAGTTGAGGTCA

### Histology and Histomorphometric Analysis

The mandibular bone and surrounding intact tissues from each euthanized animal were dissected and fixed in 4% freshly prepared paraformaldehyde (Sigma-Aldrich, St. Louis, MO) from there we used standard procedure which is previously described (Stanisic et al., 2020). In each area of interest, the total number of cells [the fibroblasts were counted manually from images captured at 60x magnification on Hematoxylin and eosin (H&E) stained sections]. All quantification and calculations of areas of interest are described previously (Stanisic et al., 2020).

### Statistical Analysis

Data analyses and graphical presentations were performed with GraphPad Prism, version 8 (GraphPad Software, Inc., La Jolla, CA). Data are represented as mean values ± standard deviation (SD) in five independent experiments in all cases. The experimental groups were compared by one-way analysis of variance (ANOVA) assuming that the values were sampled from Gaussian distributions. For a set of data, if ANOVA indicated a significant difference (*p* < 0.05); Tukey-Kramer multiple comparison tests were used to compare group means. Posttest was only performed if *p* < 0.05. If the *p* < 0.05 considered statistically significant.

## Results

### HFD Effects on the Weight Gain and Blood Glucose Levels

**Figure 2:**
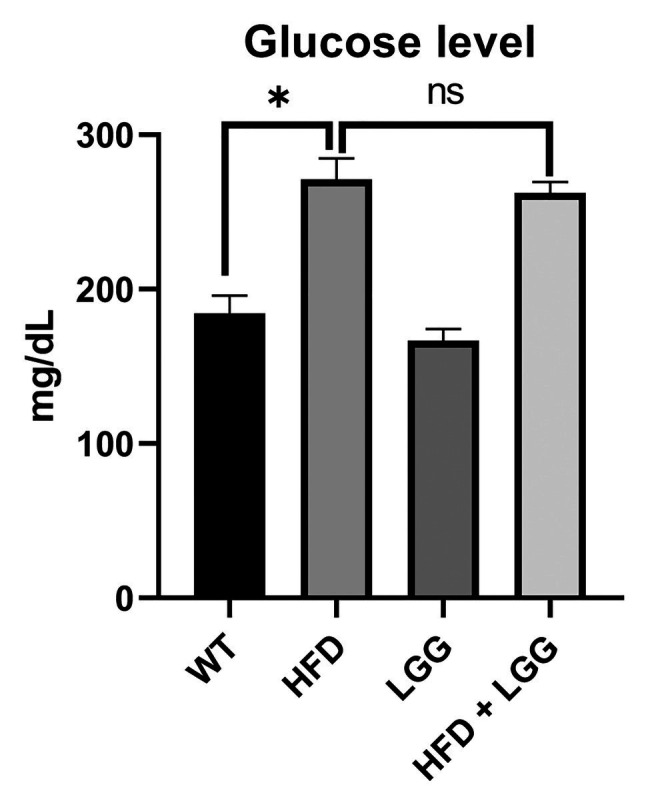
Effect of HFD and *Lactobacillus rhamnosus GG* (LGG) treatment on the glucose level (ng/ml). WT, Wild-type C57BJ/L6 mice; HFD, HFD-supplemented wild-type mice; LGG, LGG-supplemented wild-type mice; HFD + LGG, HFD, and LGG-supplemented wild-type mice. Values remain under defined diabetic values. All data represent means ± SD, WT vs. HFD group *p < 0.05; HFD vs. HFD + LGG ^ns^*p* > 0.05, *n* = 5.

Values for body weight are shown in Table 2. The significant difference in the values of these parameters was observed between groups in body weights after treatment, while no significant difference was observed in the weight before treatment. A significant increase body weight was observed in the HFD group compared to the control group (Table 2). Moreover, in the group fed with HFD increscent in blood glucose level is obvious compared to control, however, values remain under defined diabetic values and LGG treatment did not statistically affected these levels (Figure 2).

**Table 2:** Body weight measurements, before and after treatment, presented as mean ± SD.

Group	WT	HFD	LGG	HFD + LGG
BW before (g)	20.17 ± 0.5	21.27 ± 0.83	19.93 ± 0.72	21.43 ± 0.47
BW after (g)	32.93 ± 0.4*	39.33 ± 0.86*^#^	34.97 ± 0.15*	36.9 ± 0.36*

BW before vs. BW after treatment *p < 0.05; WT after vs. HFD after ^#^*p* < 0.05; *n* = 5.

### Gut Microbiota Alterations Occur Due to the HFD

**Figure 3:**
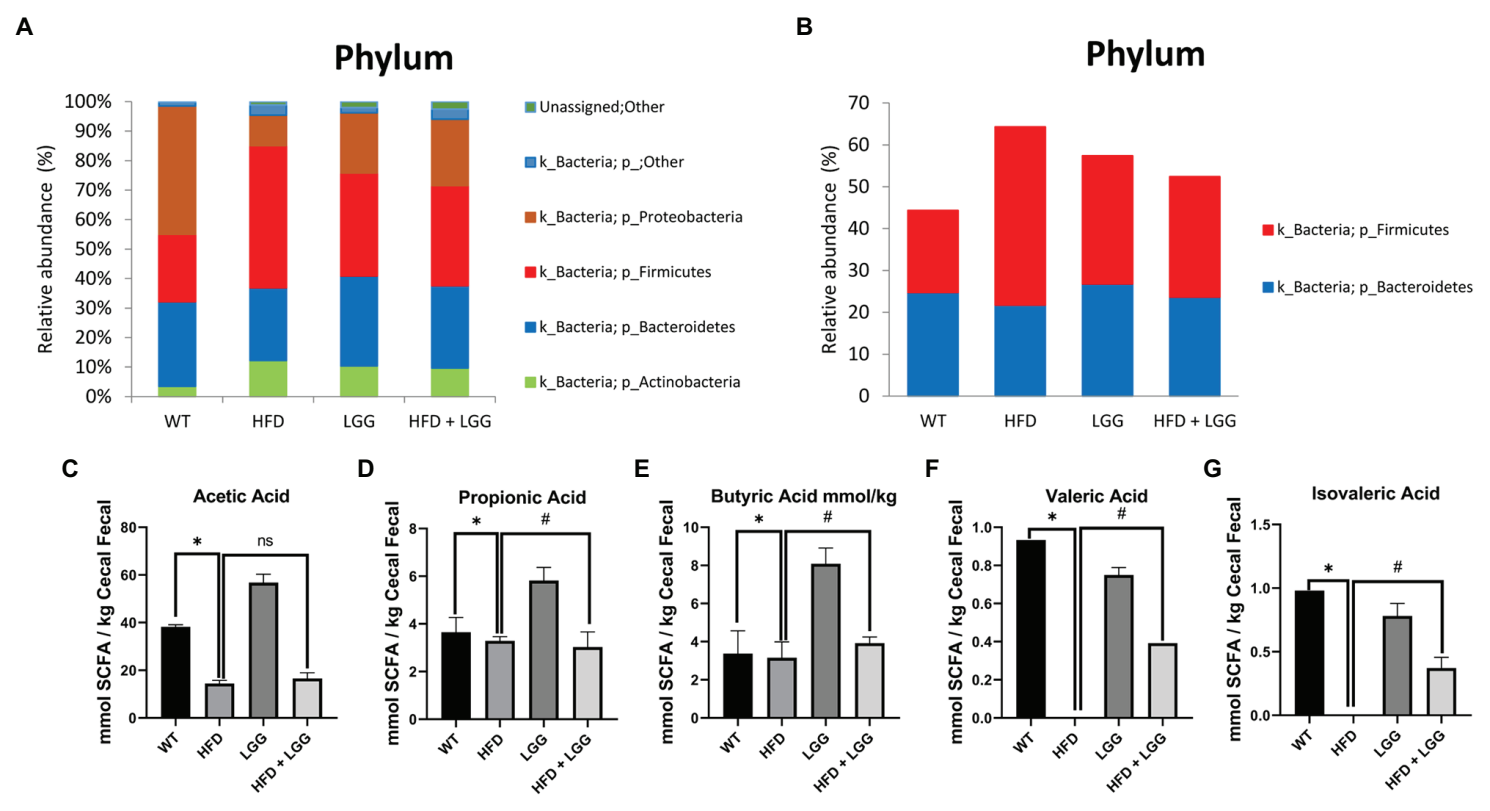
Microbiota analysis of stool in all examined groups. WT, Wild-type C57BJ/L6 mice; HFD, HFD-supplemented wild-type mice; LGG, LGG-supplemented wild-type mice; HFD + LGG, HFD and LGG-supplemented wild-type mice. **(A)** Relative abundance of total bacteria at the phylum level in each treatment group (%); **(B)** Relative abundance of Bacteroides and Firmicutes at the phylum level in each treatment group (%); **(C)** Analysis of acetic acid; **(D)** Analysis of propionic acid; **(E)** Analysis of butyrate acid; **(F)** Analysis of valeric acid; **(G)** Analysis of isovaleric acid. All data represent means ± SD, WT vs. HFD group *p < 0.05; HFD vs. HFD + LGG ^ns^*p* > 0.05, HFD vs. HFD + LGG ^#^*p* < 0.05, *n* = 5.

Deoxyribonucleic acid sequencing were done for stool samples from each of the four groups at the end of the experimental protocol to archive an overview of each phylum. We did not find a significant shift in the relative abundance of *Firmicutes* or *Bacteroidetes* in the control (relative abundance of *Firmicutes* was 19.62% and of *Bacteroidetes* was 24.73%; Figures 3A,B). However, the abundance of *Firmicutes* was increased in the HFD group (42.52%) with an accompanying decrease in *Bacteroidetes* abundance (21.74%; Figures 3A,B). Over and above, LGG treatment alone reduced the number of *Firmicutes* and increased the number of *Bacteroidetes* with especially affected HFD + LGG group where the ratio of these bacteria was almost normalized.

The results of our study showed a significant reduction in acetic (Figure 3C), propionic (Figure 3D), butyric (Figure 3E), valeric (Figure 3F), and isovaleric acids (Figure 3G) in stool samples in the HFD group (Figures 3C-G) compared to the control group. Also, a statistically significant increase in propionic, butyric, valeric, and isovaleric acids was observed in the groups treated with LGG compared to the control and HFD groups (Figures 3D-G), while values of acetic acid were not changed (Figure 3C).

### HFD Contributes Endotoxemia

**Figure 4:**
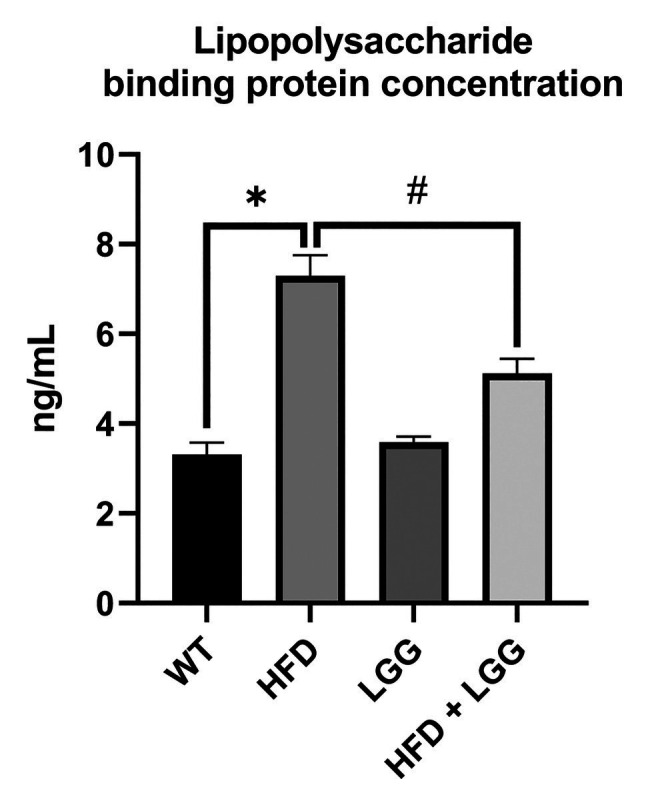
Effect of HFD and LGG treatment on the lipopolysaccharide binding protein concentration in mice serum (ng/ml). WT, Wild-type C57BJ/L6 mice; HFD, HFD-supplemented wild-type mice; LGG, LGG-supplemented wild-type mice; HFD + LGG, HFD, and LGG-supplemented wild-type mice. All data represent means ± SD, WT vs. HFD group *p < 0.05; HFD vs. HFD + LGG ^#^*p* < 0.05, *n* = 5.

Because of an HFD, there is an increased production of LPS by oral and gut bacteria. The spread of LPS through the bloodstream is made possible by LBP. Levels of LBP were measured in mice in all experimental groups. Compared to the control group, the data showed that the level of LBP significantly increased in the HFD group (*p* < 0.05). Also, data showed that the level of LBP significantly decreased in HFD + LGG group (*p* < 0.05) as compared to HFD mice (Figure 4). These results suggest that HFD, due to changes in the microbiota, affects the increased production of LPS and its spread throughout the whole body.

### HFD Causes Expression and Activity Changes in Several Inflammation Pathways in Periodontal Tissues

**Figure 5:**
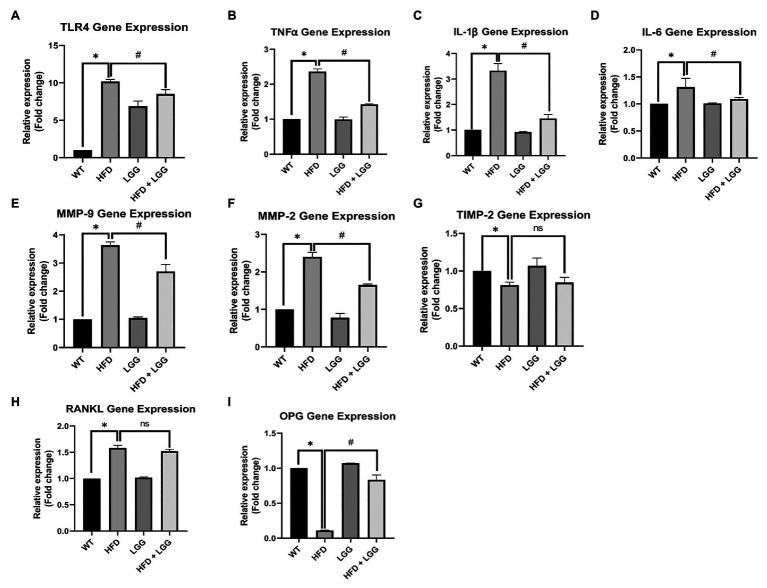
Effect of HFD and LGG treatment on the molecular remodeling in periodontal tissues. WT, Wild-type C57BJ/L6 mice; HFD, HFD-supplemented wild-type mice; LGG, LGG-supplemented wild-type mice; HFD + LGG, HFD, and LGG-supplemented wild-type mice. Quantitative RT-PCR analysis for **(A)** TLR_4_, **(B)** TNFα, **(C)** IL-1β, **(D)** IL-6, **(E)** MMP-2, **(F)** MMP-9, **(G)** TIMP-2, **(H)** RANKL, and **(I)** OPG mRNA expression. The results are presented as the expression of the individual mRNAs with normalization GAPDH, using the 2^−*Δ*ΔCq^ method. All data represent means ± SD, Fold change vs. WT group. All data represent means ± SD, WT vs. HFD group *p < 0.05; HFD vs. HFD + LGG; ^ns^*p* > 0.05, HFD vs. HFD + LGG ^#^*p* < 0.05, *n* = 5.

We measured the mRNA expression of TNFα, IL-1β, IL-6, TLR4, MMP-2, MMP-9, TIMP-2, RANKL, and osteoprotegerin (OPG). TNF-α, IL-1β, and IL-6 are the most important inflammation markers of periodontal disease, and its increase has been very well explained in periodontitis (Yucel et al., 2015) as a response to infection with the periodontal pathogens (*Porphyromonas gingivalis*, etc.). MMP-2, MMP-9, and TIMP-2 are responsible for extracellular matrix (ECM) degradation in periodontal tissue (Stanisic et al., 2019, 2020). RANKL and OPG are one of the key indicators of periodontal disease. OPG is osteoclastogenesis inhibitory factor and RANKL is an apoptosis regulator gene, a binding partner of OPG, and osteoclast differentiation factor. The data showed that TNF-α, IL-1β, IL-6, TLR4, MMP-2, MMP-9, and RANKL all peaked in periodontal tissue in the HFD group of mice. The LGG significantly decreased the expression of TNF-α, IL-1β, IL-6, TLR4, MMP-2, and MMP-9, indicating that LGG treatment attenuated the inflammation of periodontal tissue (Figures 5A-F). LGG did not affect RANKL expression (Figure 5H). OPG and TIMP-2 mRNA expression levels were significantly less in the HFD mice than WT mice (Figures 5G,I).

### HFD Contributes Matrix Degradation in Periodontal Tissue

**Figure 6:**
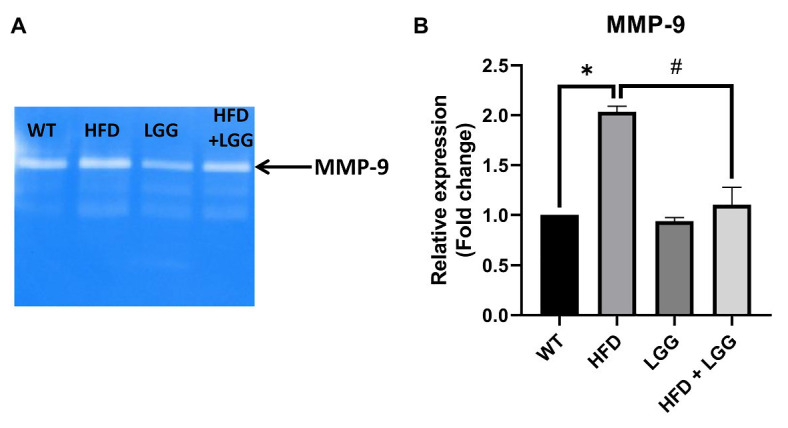
Effect of HFD and LGG treatment on the MMP activity in periodontal tissues. WT, Wild-type C57BJ/L6 mice; HFD, HFD-supplemented wild-type mice; LGG, LGG-supplemented wild-type mice; HFD + LGG, HFD and LGG-supplemented wild-type mice. **(A)** Original zymography membrane. **(B)** Gelatin zymography analysis in periodontal tissue. All data represent means ± SD, WT vs. HFD group *p < 0.05; HFD vs. HFD + LGG ^#^*p* < 0.05, *n* = 5.

**Figure 7:**
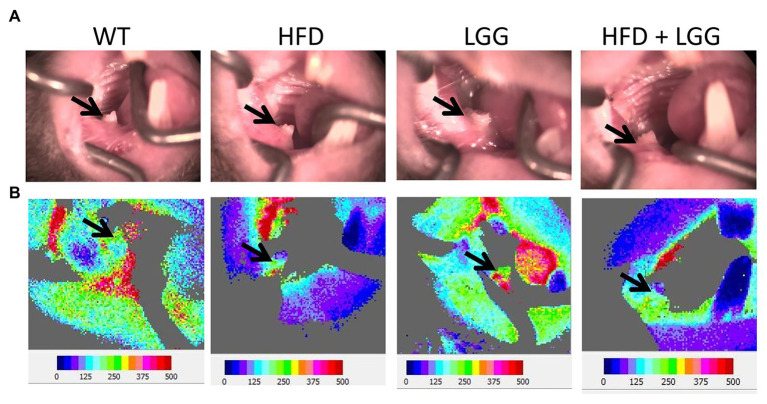
Effect of HFD and LGG treatment on the gingival blood flow. WT, Wild-type C57BJ/L6 mice; HFD, HFD-supplemented wild-type mice; LGG, LGG-supplemented wild-type mice; HFD + LGG, HFD, and LGG-supplemented wild-type mice. **(A)** Buccal view of the exposed right mandibular gingiva in first molar area. **(B)** Blood flow imaging at the surface of the buccal right mandibular gingiva in first molar area *via* Doppler laser. The black arrows indicate the gingival blood flow measurement site.

In our study, we demonstrated a significant increase in the MMP-9 activity in HFD mice in the periodontal tissue samples (Figures 6A,B). This increased MMP-9 activity provoked the degradation of matrix proteins resulting endothelial disorder of blood vessels and at the same time a disorder at the level of the periodontium, gingiva, and the alveolar bone. MMPs helped the degradation of the arterial matrix and caused decreased gingival blood flow (Figures 7A,B). The activity of MMP-9 was found to be significantly increased in the HFD mice compared to the WT and HFD + LGG groups of mice (Figure 6B).

### HFD Reduces Gingival Blood Flow

Gingival blood flow is required for conservation of periodontal tissue and tooth function. In the current study, we measured gingival blood flow (Figure 7A). Our results indicate that HFD significantly reduced blood flow (215.2 ± 10.9) in the gingiva compared to control mice (264.6 ± 9.5; Table 3). Interestingly, LGG treatment alone significantly recover gingival blood flow to basal levels which indicated the same trend in the HFD + LGG group of mice (247.7 ± 9.1), suggesting that LGG improves gingival blood flow in experimental mice treated with HFD.

**Table 3:** Gingival blood flow measurements presented as mean ± SD.

Group	WT	HFD	LGG	HFD + LGG
Flux statistic (PU)	264.6 ± 9.5	215.2 ± 10.9*	247.5 ± 8.3	247.7 ± 9.1^#^

WT vs. HFD group *p < 0.05; HFD vs. HFD + LGG ^#^*p* < 0.05; *n* = 5.

### HFD Promotes the Development of Periodontal Disease *via* Losing Alveolar Bone, Loss of Epithelial Downgrowth, and Reducing the Number of Fibroblasts

**Figure 8:**
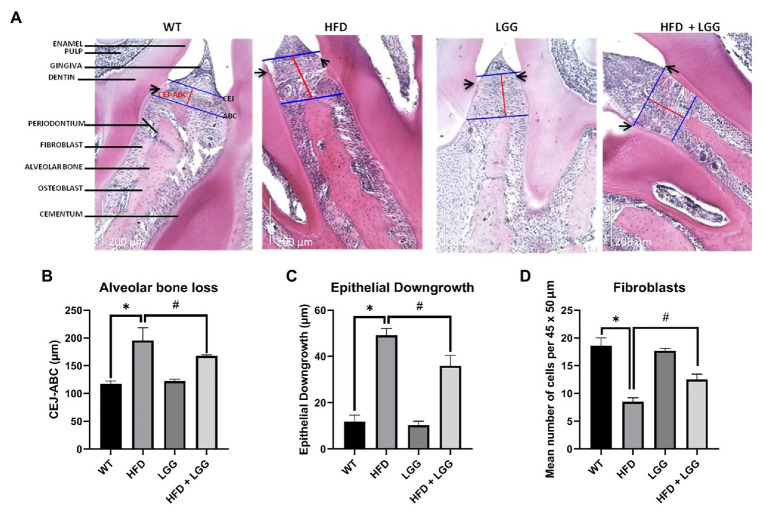
Representative images of hematoxylin-eosin (H&E) staining of periodontal tissue and histomorphometric analysis of alveolar bone loss, ED, and number of fibroblasts per 45 × 50μm. WT, Wild-type C57BJ/L6 mice; HFD, HFD-supplemented wild-type mice; LGG, LGG-supplemented wild-type mice; HFD + LGG, HFD, and LGG-supplemented wild-type mice. **(A)** Representative depiction of histological sections. Images were captured at 20× (200μm) magnification after H&E staining. Blue lines represent alveolar bone crest (ABC), and cement enamel junction (CEJ). Red lines represent the alveolar bone loss. Black arrows show the apical extent of the epithelial downgrowth (ED). **(B)** Alveolar bone-loss was measured as the distance between the CEJ and the ABC. **(C)** ED was defined by measuring the distance from the cementoenamel junction (CEJ) to the apical extent of the junctional epithelium. Results are expressed in μm. **(D)** Bars shows mean number of fibroblasts per 45 × 50μm. Scale bars = 200μm. All data represent means ± SD, WT vs. HFD group *p < 0.05; HFD vs. HFD + LGG ^#^*p* < 0.05, *n* = 5.

Periodontal tissue was stained and observed under a lens with 60x (200μm) to analyze the distance between cement enamel junction to the alveolar bone crest (CEJ-ABC), epithelial downgrowth (ED), and fibroblasts. ED was measured by the distance of the apical migration of epithelial attachment relative to the CEJ (Figure 8A). H&E staining with histomorphometric analysis showed a smaller number of fibroblasts, an increase in ED, and increased distance between CEJ-ABC in the HFD group (Figures 8B,C). Results in the HFD group showed ED, and alveolar bone-loss compared to the control group (Figures 8B,C). LGG treatment improved all the above parameters (Figures 8B,C). Our results showed a significant reduction in the number of fibroblasts in the HFD group compared to the control group (Figure 8D), which indicates destruction of periodontal tissue and promotes the development of periodontal disease.

## Discussion

Gut microbiota officiates important functions in human physiology and its composition is dependent on environmental factors, including personal hygiene, diet, drug use, and presence of toxins, as well as genetic factors (Turnbaugh et al., 2007; Lozupone et al., 2012; Jandhyala et al., 2015; Semenkovich et al., 2015; Bai et al., 2016). Alteration of the gut microbiome has been implicated in a number of diseases, including periodontitis (Greiner and Backhed, 2011; Komazaki et al., 2017; De Luca and Shoenfeld, 2019; Jia et al., 2019; Curtis et al., 2020). *Bacteroidetes* and *Firmicutes* represent two of the most important phyla which are involved in the gut microbiome. However, a shift in the proportion of bacteria *Bacteroidetes* and *Firmicutes* are associated with periodontitis (Chen et al., 2017; Yu et al., 2019). The pivotal role in the composition of the gut microbiota has diet (Scott et al., 2013). After consumption of specific dietary component, the human gut flora composition may significantly change within just a couple of days (Ng et al., 2014). Besides increscent in body weight (Table 2), long exposition to HFD has also been shown to ameliorate gut physiology, leading to changes in the composition of gut microbiota, specifically influenced by overexpression of *Firmicutes* (Turnbaugh et al., 2008). Results of our study are in correlation with previously mentioned (Figures 3A,B). We found that HFD significantly increased the total number of *Firmicutes*, but with remarkable decrease of *Bacteroidetes* (Figures 3A,B). On the other hand, LGG supplementation significantly reduced the overall abundance of *Firmicutes* while substantially increased *Bacteroidetes* (Figures 3A,B). Moreover, primates have more similar gut microbiota to human and studies of Becker et al. (2019a) on an African green monkey showed increased numbers of *Prevotellaceae* and *Bacteroidetes-Prevotella-Porphiromonas* in the established new model of Crohn’s disease. Furthermore, they investigated mutual changes in the structure of the intestine and suggested the influence of *Firmicutes*, *Bacteroidetes*, *Proteobacteria*, *Archaebacteria*, and *Actinomycetes* on cytokines and its mediators (Becker et al., 2019b). Those observations were in consistence with the results of our study (Figures 3A,B). It still remains unclear how this enrichment influences periodontitis state, making this topic at the high range of interest in scientific world. Taking everything above into consideration, we can possibly say that even small classes and bacteria can have an impact on microbiome in such an essential way that those effects will not be just reflected at the phyla level.

The change in the microbiota composition gives different amounts of metabolites that are produced in the gut (Yang et al., 2019). SCFA are one of the major categories of metabolites produced in the gut. Gut microbiota produces the following SCFA, butyrate, acetate, propionate, valeric, and isovaleric acid (Lu et al., 2014). SCFA play significant roles in many diseases and metabolic disorders such as diabetes (Tolhurst et al., 2012) and obesity-related inflammation (Al-Lahham et al., 2011). Moreover, fermentation of anaerobic bacteria can lead to production of SCFA and might establish themselves in places which are associated with pathology such as periodontal disease (Niederman et al., 1997; Tsuda et al., 2010). The results of our study had shown a significant reduction in acetic, propionic, butyric, valeric, and isovaleric acids in fecal samples in the HFD group compared to the control group. Also, a significant increase in propionic, butyric, valeric, and isovaleric acids was observed in the groups treated with LGG compared to the control and HFD groups (Figures 3D-G), while acetic acid was not significant altered (Figure 3C). This observation suggests that, in our case, HFD through reduction of SCFA can support the reduction of gingival blood flow and the occurrence of periodontal disease (Figures 1, 7; Table 3). Furthermore, in gingival crevicular fluid of patients with chronic periodontitis low levels of propionic and butyric acids were found (Niederman et al., 1997; Qiqiang et al., 2012), and this can prevent tight-binding potential of epithelial cells resulting in penetration of bacterias and destruction of periodontal tissue (Takigawa et al., 2008). Periodontopathogen bacteria is responsible for the production of butyric acid which is mainly involved in the development of periodontal disease onset (Cueno and Ochiai, 2018). Interestingly, direct application of butyric acid in gingival tissue causes activation of NF-κB in the blood only after 60 to 180min (Cueno et al., 2016), indicating its short-term immediate effect. Also, Cueno and Ochiai (2018) studies indicate that elevated values of butyric acid in the gingiva caused the quantity of representative inflammatory markers (Caspases 12 and 1, IL-1β, and TNF-α) to decrease. The results of our study prove precisely that SCFA (Figures 3C-G), reduction increases inflammatory markers such as IL-1β and TNF-α in periodontal tissue (Figures 5B,C). However, the effect of gut butyric acid and periodontal disease has not been sufficiently elucidated and investigated. The reduction of SCFA in the stool affected gut permeability and consequently helped to change the gingival blood flow and the occurrence of inflammation of periodontal tissue. Additional research on the effects of individual action of gut SCFA and periodontal disease is needed to elucidate the mechanisms of their action. In summary, we can observe that HFD can support the reduction of gingival blood flow and the occurrence of periodontal disease through reduction of SCFA.

Live bacterias and LPS as bacterial component, can change location from the gut lumen to other tissue sites (Scheithauer et al., 2016). LPS, as the most important component of the Gram-negative bacteria membrane, binds to Toll-like receptor 4 (TLR4) and activates innate immune response. In our study, elevated level of this receptor expression was observed in the HFD group (Figure 5A; Wei et al., 2020). LPS transfer to the TLR4 is catalyzed by local and systemically LPS binding protein. Furthermore, hepatocytes are responsible for the production of LBP which blood levels are increased in chronic and aggressive periodontitis (Soolari et al., 1999; Wohlfeil et al., 2012). We measured the level of LBP in the mice serum in order to detect another potential route of periodontal disease (HFD gut dysbiosis-LPS/TLR4 pathway-gingival blood flow-periodontal disease). The results of our study showed that LBP was significantly increased in HFD group compared to the control group, while in the HFD + LGG group a decrease of LBP levels compared to the HFD group was observed (Figure 4).

According to a recent study, we also have investigated the role of HFD during inflammation and its possibility to cause periodontal tissue damage (Muluke et al., 2016). The expression of TNFα, IL-1β, IL-6, TLR4, RANKL, and OPG in periodontal tissue of mice was measured. HFD promotes the development of gingivitis and periodontitis by systemic and local inflammatory response. Blasco-Baque et al. (2012) showed that TNF-α, IL-1β, and IL-6 periodontal tissue expression were increased in the periodontal tissue of HFD mice. It has been shown that inflammatory cytokines such as TNF-α, IL-1β, IL-6 are in tight correlation with development of periodontal disease (Pacios et al., 2012; Cekici et al., 2014). Jin et al. (2013) showed LPS/TLR4 pathway might have a pivotal role when it comes to exposure of macrophages to LPS due to palmitic acid which may provoke increased production of inflammatory cytokines specifically through this pathway. Our study of periodontal tissue overlaying areas of alveolar bony defects revealed that HFD animals indeed exhibited higher expression of TNF-α, TNFα, IL-1β, IL-6, TLR4, and RANKL (Figures 5A-D,H) and lower expression of OPG (Figure 5I) compared with control animals, suggesting that HFD promotes local inflammatory activation in response to infection.

Elevated proinflammatory cytokines production induces increased activity and expression of MMP. Previously conducted experimental studies have found that HFD causes loss of vascular elasticity, primarily due to deposition of harmful substances in the walls of blood vessels, which in turn leads to reduction of blood flow (Declèves et al., 2013; Mitra et al., 2018; Yuan et al., 2019). Since MMPs are one of the factors causing the occlusion as well as damage of blood vessels and periodontal tissue, MMP-2, MMP-9, and TIMP-2 expression was measured in periodontal mice tissue. MMPs are responsible for ECM degradation catalyzed by zinc-dependent endopeptidases. MMPs can selectively digest pathological ECMs in different models of disease or injury. Additionally, improper digestion of ECMs can lead to fibrosis in different tissues (Chen and Li, 2009). The results of this study showed that MMP-2 and MMP-9 were significantly increased in the HFD group, while the HFD + LGG group reduced mRNA levels from MMPs (MMP-2 and MMP-9) compared to HFD groups (Figures 5E,F). Moreover, it was noticed that the expression and activity of MMP-9 were increased in experimental periodontitis, regardless of the method of periodontitis induction (Barreiros et al., 2018). These studies indicated the importance of ECM degradation as one of the factors in the periodontal disease development. In our study, MMP-9 activity was measured by gelatin zymography in periodontal mice tissue. The results showed that MMP-9 activity was significantly increased in the HFD group, while the LGG treatment reduced the level of MMP-9 activity compared to the HFD group (Figure 6A).

Previously conducted study had shown that HFD causes endothelial damage after 2h of meal *via* vascular occlusion, leading to a reduction in blood flow, which is consistent with our results (Bui et al., 2010; Münch et al., 2019). We used HFD to determine its effect on periodontal tissues, which can be direct on the oral cavity, or indirect on the blood vessels, blood flow, and gastrointestinal tract (Figure 1). Blood flow in the gingiva indicates circulation in the gingival and periodontal tissue and their nutrition and function. We measured gingival blood flow by using Laser Doppler. In the buccal gingiva region of the first right mandibular molar, GBF was 215.2 ± 10.9 flux units in the HFD group, while in the control group it was 264.6 ± 9.5 flux units (Table 3). Because of HFD, there was a reduction in gingival blood flow while LGG in the HDF + LGG group improved gingival blood flow (247.5 ± 9.1 flux unit). For the first time in our study, it has been shown the influence of HFD on gingival blood flow in mice (Figures 7A,B). Baab and Oberg (1987) suggested that some significant morphological and changes in vascularization occurred as an early sign of gingivitis in dogs while measured blood flow in the inflamed gingiva did not show very convincing evidence. Furthermore, in previous experimental studies, it was hypothesized that there are three predisposing factors for gingivitis occurrence, including vascular stasis, stress, and smoking. These studies also found a reduction in the gingival blood flow during ischemia in rabbits infused with epinephrine and nicotine (Clarke et al., 1981; Clarke and Shephard, 1984).

Numerous disorders lie in the pathology of periodontal disease development. Firstly, HFD causes periodontal disease (Blasco-Baque et al., 2012), which was also confirmed in our study (Figures 8A-D). We used histomorphometric analysis as reported in previous studies (Semenoff et al., 2008). ED and CEJ-BC were chosen thus their previous correlation with attachment loss and bone-loss (Susin and Rösing, 2003; Breivik et al., 2006). Histomorphometric analysis revealed an increase in the distance of CEJ-ABC in the HFD group, which indicates the existence of periodontal disease (Figure 8B). For histomorphometric analysis, the mean distance from the CEJ-ABC and the mean ED in μm was statistically significantly higher in the HFD mice group (*p* < 0.05 vs. WT; Figures 8A-C). Based on studies of similar design and obtaining the values for the distance CEJ-ABC, we can conclude that periodontal disease exists in our mice. In our study, we discovered the difference in periodontium between these four groups of mice, and the influence of HFD and dysbiosis on the periodontium, as well as the occurrence of periodontal disease. HFD mice showed an increase in the periodontal breakdown by molecular remodeling (Figures 5A-I), matrix degradation (Figures 6A,B), decreased gingival blood flow (Table 3), alveolar bone loss (Figure 8B), and decreased number of fibroblasts (Figure 8D). These findings as well are in correlation with previously described. Decreased fibroblast levels were observed in the experimental model of periodontitis in mice compared to healthy controls from wild type group (Alshammari and Amar, 2019).

Our hypothesis was established on the effects of HFD in sense of inducing chronic inflammation throughout increscent of TNF-α, IL-1β, and IL-6 levels and on such a way initiating destruction of the periodontium thus reduced the number of fibroblasts. In addition to the above, increased expression of organic acids might be interpreted as the important factor for suppressing nuclear factor kappa-*B* and inflammation overall. On the other hand, HFD, by suppressing this pathway leads to more intense inflammation and periodontal disease.

The results of this study indicate for the first time the influence of HFD and dysbiosis on gingival blood flow. Also, the results of this study suggest that oral LGG therapy may regulate gingival blood flow. With this study, we provided theoretical evidence for the dysbiotic mechanism of decreased blood flow and their impact on the development of periodontal disease as well as support for the treatment of LGG gingival blood flow. However, the specific mechanisms and clinical effect of HFD and LGG on periodontal disease require further research.

## Data Availability Statement

The raw data supporting the conclusions of this article will be made available by the authors, without undue reservation.

## Ethics Statement

The animal study was reviewed and approved by the Institutional Animal Care and Use Committee (IACUC #19592) of the University of Louisville, KY.

## Author Contributions

The authors confirm that they are the original contributors of this work and all of them approved it for its publication.

### Conflict of Interest

The authors declare that the research was conducted in the absence of any commercial or financial relationships that could be construed as a potential conflict of interest.

## Abbreviations

ABC: Alveolar bone crest
CEJ: Cement enamel junction
DNA: Deoxyribonucleic acid
ECM: Extracellular matrix
H&E: Hematoxylin and eosin
HFD: High fat diet
IL-1β: Interleukin-1 beta
IL-6: Interleukin-6
LBP: Lipopolysaccharide binding protein
LGG: *Lactobacillus rhamnosus GG*
LPS: Lipopolysaccharide
MMP: Matrix metalloproteinase
OPG: Osteoprotegerin
PBS: Phosphate-buffered saline
*P. gingivalis*: *Porphyromonas gingivalis*
RANKL: Receptor activator of nuclear factor kappa-*Β* ligand
RNA: Ribonucleic acid
RT-PCR: Real-time polymerase chain reaction
SCFA: Short chain fatty acids
TLR4: Toll-like receptor 4
TNF-α: Tumor necrosis factor alpha
